# Chlorothalonil: an effective bacteriostatic agent for bud induction of *Acacia auriculiformis* under open condition (non-axenic)

**DOI:** 10.1186/s13007-019-0390-3

**Published:** 2019-01-25

**Authors:** Liejian Huang, Hong Wang, Muhammad Qasim Shahid, Bingshan Zeng

**Affiliations:** 1 Research Institute of Tropical Forestry, CAF, 682# Guangshan 1 Road, Guangzhou, 510520 Guangdong China; 2 State Key Laboratory for Conservation and Utilization of Subtropical Agro-Bioresources, South China Agricultural University, Guangzhou, 510642 China; 3 College of Agriculture, South China Agricultural University, 483# Wushan Road, Guangzhou, 510642 Guangdong China

**Keywords:** Bacteriostatic agent, Chlorothalonil, Bud induction, Open condition, *Acacia auriculiformis*

## Abstract

**Background:**

Open tissue culture technique could be simplified by using different bacteriostatic agents. There is a great difference in the bacteriostatic effects of different antimicrobial agents on various explants. However, there is no report about the effective bacteriostatic agent for open tissue culture of *Acacia auriculiformis*.

**Results:**

We carried out the bud induction trials under open conditions to screen out an effective antibacterial agent for open tissue culture of *A. auriculiformis*. The results showed that the suitable type and concentration of bacteriostatic agent was 0.2 g L^−1^ Chlorothalonil, and the suitable explant type was middle shoot section with leaves (the shoot section with third to fifth axillary bud). The treatment of 0.8 g L^−1^ Carbendazim for 3 min was the most suitable strategy for explants disinfection, and October was the best time for explants collection. The **s**uitable bud induction medium was 1/8 MS + agar 7 g L^−1^ + Chlorothalonil 0.2 g L^−1^ + 6-BA 1.5 mg L^−1^, and the bud induction rate was 99.54%.

**Conclusions:**

Our results revealed that Chlorothalonil is an effective bacteriostatic agent for bud induction of *A. auriculiformis* under open condition. These results would be very helpful for further establishment of open tissue culture technology for *A. auriculiformis*.

## Background

The technique of tissue culture plays an important role in tree breeding, and we can produce a large number of seedlings in limited space using this technique while maintaining excellent characters of the elite plants. However, high temperature and high pressure are usually necessary for sterilization during the process of tissue culture, and seedlings are apt to be polluted. So, the cost of seedling production by tissue culture technique is high.

In order to reduce the costs of the seedling production, researchers have tried to find out a new technique of tissue culture that easy to operate with low pollution rate. Herman pointed out that some plants could be cultured under the non-axenic conditions [1]. According to the Herman’s opinion, some researchers proposed a new tissue culture method that culture the plantlets under open conditions with an appropriate bacteriostatic agent and no need of a strict aseptic technique, and is called as open tissue culture method. During the process of open tissue culture, the bacteriostatic agent directly acts on the plantlets, which could inhibit endophytic fungi effectively than using traditional tissue culture method. Moreover, there is no need to sterilize with high temperature and high pressure and no need of an ultra-clean bench by using this method. Therefore, this method simplified the tissue culture technique, and reduces the cost of seedlings production [2–4].

*Acacia auriculiformis* is one of the most important *Acacia* species in China because of so many advantages, such as fixing-N, improving soil, and suitable for making paper and furniture. But at present, the development of *A. auriculiformis* plantation is greatly affected by the lack of perfect asexual propagation technique. So, it is very important to establish a perfect asexual propagation technique and to reduce the cost of seedlings production. For different explants, the bacteriostatic effects of antimicrobial agents varied significantly [5–11]. In addition, there is little known about the effective bacteriostatic agent for open tissue culture of *A. auriculiformis*.

Bud induction is a key step in the process of tissue culture, not only for traditional tissue culture, but also for new method of tissue culture. When buds are successfully induced, they can produce reproductive material for subsequent proliferation and rooting. However, there is no easy way to induce the buds because bud induction is affected by various factors such as the types of explants, collection season, sterilization treatment methods, and induction culture medium. Therefore, a simple and effective bud induction technique is important for the establishment of tissue culture under open conditions.

In this study, *A. auriculiformis* was used as a material for bud induction. We successfully established a bud induction technique for *A. auriculiformis* under open conditions by using different types and concentrations of antimicrobial agents, explant types, disinfection methods, bud induction mediums and collection seasons. Our results provided an important foundation for further establishment of open tissue culture technique for *A. auriculiformis*. We screened out the proper bacteriostatic agent to simplify tissue culture technique of *A. auriculiformis* under open conditions, which could considerably reduce the costs of tissue culture. The results are important for the breeding and application of *A. auriculiformis*, and would provide an important reference for other tree species.

## Results

### The types and concentrations of antimicrobial agents

Pollution rates were higher for the Carbendazim treatment than Chlorothalonil when the two antibacterial agents were used in the same concentrations (Table 1), which suggests that Carbendazim is not suitable as an antibacterial agent for *A. auriculiformis*. Browning rates increased as Chlorothalonil concentration increased, and survival rates significantly reduced at high concentrations of Chlorothalonil. The survival rate of *A. auriculiformis* explants was 83.33% at 0.2 g L^−1^ concentration of Chlorothalonil. Therefore, 0.2 g L^−1^ of Chlorothalonil was considered as a suitable concentration for *A. auriculiformis*.

**Table 1:** Effects of bacteriostat agents on bud induction of *Acacia auriculiformis* under open condition

Bacteriostat	Concentration (g L^−1^)	Browning rate (%)	Pollution rate (%)	Survival rate (%)
Chlorothalonil	0.8	69.45a ± 8.68	15.28bc ± 2.41	15.28d ± 6.37
	0.6	30.56b ± 2.40	16.67bc ± 15.02	52.74bc ± 13.39
	0.4	23.61bc ± 4.82	13.89bc ± 2.41	62.50bc ± 7.22
	0.2	11.11de ± 2.41	5.56c ± 2.40	83.33a ± 4.17
Carbendazim	0.8	23.61bc ± 2.41	16.67bc ± 4.17	59.72bc ± 6.37
	0.6	18.06 cd ± 2.40	19.44bc ± 2.40	62.50bc ± 4.17
	0.4	6.94e ± 2.40	26.39b ± 2.41	66.67b ± 4.17
	0.2	9.72de ± 2.41	44.44a ± 6.36	45.83c ± 7.22
Control	0.0	2.78e ± 2.41	55.55a ± 4.17	41.67d ± 4.17

Different letters indicate significant differences at P < 0.05. ± standard deviation

### The types and disinfection of explants

Browning rate, pollution rate and survival rate differed significantly among different types of explants, and the explants of middle shoot section with leaves were found to be the best (Table 2). The browning rates were significantly higher in the explants of upper shoot section with leaves and the middle shoot section without leaves than the explants of middle shoot section with leaves, regardless of the method of disinfection. Moreover, the survival rates were significantly lower in the explants of upper shoot section with leaves and the middle without leaves than middle shoot section with leaves.

**Table 2:** Effects of explant type and disinfection strategy on bud induction of *Acacia auriculiformis* under open condition

Explant type	Disinfection strategy	Browning rate (%)	Pollution rate (%)	Survival rate (%)
Upper shoot section with leaves	0.8 g L^−1^ Carbendazim for 1 min	38.10c ± 0.00	14.29a ± 9.52	47.62c ± 9.52
	75% Alcohol for 2 s	85.71a ± 4.77	4.76ab ± 0.00	9.52d ± 4.76
	75% Alcohol for 5 s	90.48a ± 4.77	4.76ab ± 4.76	4.76d ± 4.76
Middle shoot section with leaves	0.8 g L^−1^ Carbendazim for 3 min	0.00e ± 0.00	0.00b ± 0.00	100a ± 0.00
	75% Alcohol for 10 s	14.28d ± 12.60	14.29a ± 4.77	71.42b ± 9.52
	75% Alcohol for 15 s	19.05d ± 0.00	9.52ab ± 0.00	71.43b ± 0.00
Middle shoot section without leaves	0.8 g L^−1^ Carbendazim for 3 min	52.38b ± 4.76	9.52ab ± 4.77	38.10c ± 9.52
	75% Alcohol for 10 s	57.14b ± 16.49	4.76ab ± 0.00	38.10c ± 16.49
	75% Alcohol for 15 s	66.67b ± 8.25	4.76ab ± 4.76	28.57c ± 9.53

Different letters indicate significant differences at P < 0.05. ± standard deviation

The survival rate of *A. auriculiformis* was 100.00% when middle shoot section with leaves was used as an explant and 0.8 g L^−1^ Carbendazim treated for 3 min for disinfection, which produced significantly higher survival rate than other treatments. Therefore, the middle shoot section with leaves as an explant, and 0.8 g L^−1^ Carbendazim for 3 min was considered as an optimal treatment for explants.

### Medium of bud induction

For bud induction of *A. auriculiformis*, both browning and pollution rates were zero when no nutrient element was added into the medium, but bud induction rate was not so good (Table 3). Browning and bud induction rates were higher in 1/8 MS than non-nutritive medium, although significant difference was only found for bud induction rate. Browning rate increased but bud induction rate decreased sharply as nutrient elements increased. For 1/8 MS medium, the bud induction rate increased significantly with 6-BA concentration, and bud induction rate was the highest (86.83%) at 1.0 mg L^−1^ concentration of 6-BA. Therefore, 1/8 MS was found to be the best bud induction medium for *A. auriculiformis*.

**Table 3:** Effects of medium type and 6-BA on bud induction of *Acacia auriculiformis* under open condition

Treatment		Browning rate (%)	Pollution rate (%)	Survival rate (%)	Bud induction rate (%)
Medium type	6-BA (mg L^−1^)				
Control	0	0.00e ± 0.00	0.00a ± 0.00	100.00a ± 0.00	48.61c ± 8.67
	0.5	0.00e ± 0.00	0.00a ± 0.00	100.00a ± 0.00	50.00c ± 4.17
	1.0	0.00e ± 0.00	0.00a ± 0.00	100.00a ± 0.00	52.78c ± 2.41
1/8 MS	0	1.39e ± 2.41	0.00a ± 0.00	98.61a ± 2.41	43.47c ± 12.04
	0.5	1.39e ± 2.41	1.39c ± 2.41	97.22a ± 4.81	63.00b ± 4.94
	1.0	5.56e ± 2.41	0.00a ± 0.00	94.44a ± 2.40	86.83a ± 4.15
1/4 MS	0	59.72abcd ± 14.63	11.11abc ± 10.49	29.17 cd ± 21.65	0.00e ± 0.00
	0.5	16.67e ± 28.87	1.39c ± 2.41	81.94a ± 31.28	3.03e ± 5.25
	1.0	12.50e ± 7.22	1.39c ± 2.41	86.11a ± 6.36	19.09d ± 9.05
1/2 MS	0	66.67abc ± 8.34	11.11abc ± 4.81	22.22 cd ± 12.73	0.00e ± 0.00
	0.5	48.61 cd ± 19.69	8.33bc ± 4.17	43.05bc ± 20.97	0.00e ± 0.00
	1.0	31.95de ± 4.81	0.00a ± 0.00	68.05ab ± 4.81	10.32de ± 3.93
Modify MS	0	79.17ab ± 11.02	18.06a ± 6.36	2.78d ± 4.81	0.00e ± 0.00
	0.5	87.50a ± 4.17	12.50ab ± 4.17	0.00d ± 0.00	0.00e ± 0.00
	1.0	54.17bcd ± 27.32	1.39c ± 2.41	44.44bc ± 27.74	11.11de ± 11.11

Different letters indicate significant differences at P < 0.05. ± standard deviation

**Fig. 1:**
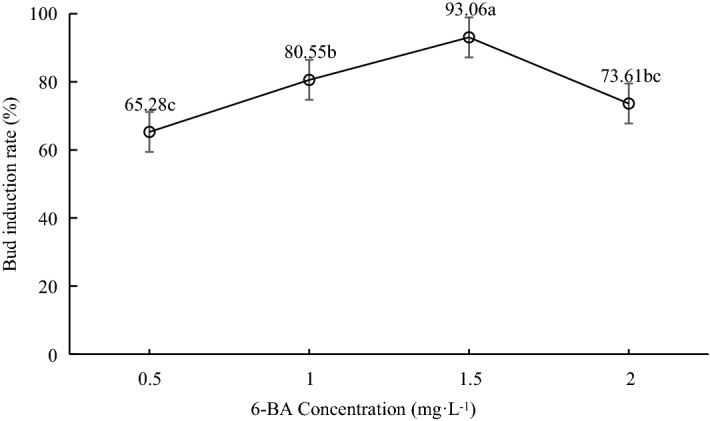
Effects of 6-BA on bud induction rate of *Acacia auriculiformis* under open condition. Lower case letters indicate significant differences at P < 0.05. Vertical bars indicate standard deviation

Although bud induction rate increased as 6-BA concentration increased, it wasn’t clear about the optimum concentration of 6-BA. Therefore, we used four different concentrations of 6-BA, including 0.5 mg L^−1^, 1.0 mg L^−1^, 1.5 mg L^−1^ and 2.0 mg L^−1^, and the results are shown in Fig. 1. In 1/8 MS medium, when 6-BA concentration increased from 0.5 to 1.5 mg L^−1^, the bud induction rate improved significantly. However, the bud induction rate was decreased from 93.06% to 73.61% with the further increase in 6-BA concentration from 1.5 to 2.0 mg L^−1^. Therefore, the optimum bud induction medium for *A. auriculiformis* was 1/8 MS + 6-BA 1.5 mg L^−1^.

### Season of explants collection

The bud induction rate of explants collected in January was significantly lower than those collected in April, July and October (Table 4). For *A. auriculiformis*, October was the best season for explants collection as indicated by the highest bud induction rate (99.54%) than those collected in other seasons.

**Table 4:** Effects of explants collection season on bud induction of *Acacia auriculiformis* under open condition

Seasons	Bud induction rate (%)
January	47.68b ± 2.12
April	98.15a ± 1.60
July	97.68a ± 0.80
October	99.54a ± 0.80

Different letters indicate significant differences at P < 0.05. ± standard deviation

## Discussion

### The types and concentrations of antimicrobial agents

Because of the addition of antimicrobial agents in medium, the open tissue culture does not require a strict aseptic environment such as high temperature, high pressure sterilization, and super clean bench. So, the screening of suitable antimicrobial agents is very important for open tissue culture.

Sodium hypochlorite was suitable for open tissue culture of *Broussonetia papyrifera* hybrid (10 mg L^−1^ sodium hypochlorite) [12] and tobacco (1 g L^−1^ sodium hypochlorite) [6], while Carbendazim was suitable for cherry root stocks [7]. The effect of manganese zinc was better than that of thiophanate methyl for open tissue culture of potato [13]. Carbendazim and Mancozeb were appropriate for *Amygdalus pedunculata* [8], while H189 was considered as suitable for grape open tissue culture [9]. Cui et al. [11] revealed that the biological components extracted from many plants have a certain inhibitory effect on endophytes and less damage to explants viz. high affinity with plant tissues and easy to penetrate into the plant. However, this kind of bacteriostatic agent was very difficult to obtain. Carbendazim could not inhibit micrococcus and Penicillium, but Chlorothalonil could only inhibit *Aspergillus niger* [10]. The previous studies revealed that bacteriostatic effects of antimicrobial agents varied significantly among different types of explants/plants.

In this study, we selected readily available and broad-spectrum antibacterial agents, Chlorothalonil and Carbendazim, for bud induction under open conditions in *A. auriculiformis*. The results showed that Chlorothalonil had better bacteriostatic effects, and the suitable concentration in culture medium was 0.2 g L^−1^, which effectively controlled the pollution rate (less than 5%), and successfully induce bud under open conditions. Our results were not consistent with the results of Zhang et al. [10], who revealed that Chlorothalonil could only inhibit *Aspergillus niger*, which might be happened due to difference in explants used in both studies.

### Selection and treatment of explants

In the open tissue culture, the physiological condition of the explants should be good because bacteriostatic agents have strong effect on explants. The type and length of explants have certain influences on bud induction. Healthy explants with good physiological conditions can greatly improve the success rate of bud induction under open condition.

We found that the semi-lignified sections from middle part of the shoots were the best as explants, which was consistent with the previous studies [14–16]. About 75% of the nutrients required for root growth and development are derived from old leaves, which showed that the proper preservation of old leaves on cuttings is beneficial for rooting [17]. We used three types of explants based on the results of previous studies, i.e. middle shoot section with and without leaves, and upper shoot section with leaves, and obtained suitable explant type under open conditions. Because different species and explants required different disinfection strategies, and the selection of disinfection strategy must be combined with the selection of explants.

Mercuric chloride is usually used to sterilize explants, but it may cause serious injury to explants and bud induction rate might be reduced. Pretreatment of Rifampicin solution on *Carica papaya* sprout effectively reduced the pollution rate, and no toxic effect was found [18]. A mixture of Carbendazim, polyvinylpyrrolidone, ascorbic acid, citric acid and benzyl penicillin as the pretreatment agent reduced the pollution rate of *Madhuca hainanensis* explants [19]. However, all these antimicrobial agents are not universal for all tree species, and it is necessary to screen out the suitable antimicrobial agent for different species. Here, we used middle shoot sections with leaves as explants, and soaked the explants in 0.8 g L^−1^ Carbendazim for 3 min, and obtained good bacteriostatic effects and survival rate was as high as 100%. This method can be used for open tissue culture of *A. auriculiformis* under open conditions.

### Bud induction of *Acacia auriculiformis*

Using explants from adult trees for tissue culture is useful to accelerate tree breeding program and maintain good characteristics of elite tree. But bud induction of the explants from adult tree is difficult because of the maturity effect [20]. The composition of culture medium affects the browning of explants. Low concentration of inorganic salt is conducive to decrease browning, and high concentration of inorganic salt can increase browning [20]. But there is a great variation in the response of different tree species to culture medium. For the same inorganic salt, high concentrations may lead to browning for some tree species, but may reduce browning rate for other tree species. For example, the browning rates were low when 1/2 DKW or 1/2 MS (low salt concentration) were used as culture medium in *Annona* [21], and *Spathiphyllum kochii* [22], respectively. Similarly, a high salt concentration medium inhibited browning in *Ginkgo biloba* [23] and *Rubus fruticosus* [24].

Here, we used a different medium for open bud induction of *A. auriculiformis* compared to the traditional tissue culture method. In a previous study, the best bud induction medium for *A. auriculiformis* was MS + 6-BA 1.0 mg L^−1^ + sucrose 40 g L^−1^, which produced a bud induction rate of 92% [20]. In this study, *A. auriculiformis* had high browning rates when the mediums were modified MS, 1/2 MS and 1/4 MS. No obvious changes in explants were observed at the initial stage of inoculation, but the explants were almost completely brown after culture for 10 d. The antimicrobial agent added in medium may react with certain substances in MS, which lead to the browning of explant, but it needs further studies to verify this phenomenon. Our results revealed that low nutrient contents in medium favored bud induction of *A. auriculiformis* under open conditions, and the suitable medium for bud induction of *A. auriculiformis* was 1/8 MS. Although there was no sugar in the culture medium, the explants mainly depend on the availability of light to maintain vitality and budding.

The optimum concentrations of 6-BA for bud induction under open condition were also different from those in traditional culture methods. 1.5 mg L^−1^ 6-BA was optimum concentrations for bud induction under open conditions for *A. auriculiformis*, which was higher than the optimum concentrations found in traditional tissue culture (1.0 mg L^−1^ 6-BA) [20]. Overall, higher concentrations of 6-BA were required for bud induction of *A. auriculiformis* under open conditions than in traditional culture, possibly antibacterial agents in mediums affect the effectiveness of 6-BA. Moreover, the bud induction rate of explants collected in October was the highest, while it was the lowest in January when the temperature was low. These results indicated that temperature may have a significant effect on explants collection but it requires further studies.

Here, the bud induction rates of *A. auriculiformis* reached to 99.54% under open condition, which was higher than those obtained by traditional tissue culture (92.00%) [20]. The reason may be that bacteriostatic agents lowered pollution rate and raised the survival rate and the utilization ratio of explants. There were no obvious differences in germination time and growth rate of axillary bud between open tissue culture and traditional tissue culture. Axillary bud germination occurred at around 10 d, and axillary bud grew to about 2 cm after 30 d for both open tissue culture and traditional tissue culture. Therefore, open tissue culture is better than traditional tissue culture because former promotes bud induction rate.

## Conclusion

We selected the branches from 18-year-old elite plants for roots development to establish the cutting orchard. Explants were selected from the annual cutting orchard and immediately treated in the following steps: after cleaning with a soft brush, explants were soaked in washing powder water for 30 min, and rinsed with water for 10 min. Then we cut the middle section of shoot explant (third to fifth axillary bud) with half leaves for 2–3 cm, followed by soaking into 0.8 g L^−1^ Carbendazim for 3 min, and then inoculated in the medium. The results showed that this pretreatment method can control pollution and have better inhibitory effect on endophytic bacteria. The suitable antibacterial agent for *A. auriculiformis* was Chlorothalonil, and the suitable concentration was 0.2 g L^−1^ for effective control of pollution and high survival rates (83.33%). October was found as the most suitable season for explant collection. The **s**uitable bud induction medium was 1/8 MS + agar 7 g L^−1^ + Chlorothalonil 0.2 g L^−1^ + 6-BA 1.5 mg L^−1^ for *A. auriculiformis*. Bud induction rate was close to 100% for *A. auriculiformis* under open condition. The results indicated that using suitable antibacterial agent was beneficial for bud induction of *A. auriculiformis* under open conditions, and antibacterial agents didn’t prevent explant growth. Only low quantity of nutrient elements was required for suitable medium, which would greatly save the costs of medium consumption, and thus reduce the costs of tissue culture. In short, by using bud induction culture under the open conditions, bud induction rate and efficiency improved significantly, and the time and material costs reduced as well.

## Methods

### Materials

The shoots from 1-year-old cutting orchard with good growth status and full of axillary buds were selected in this study according to Shi et al. [15] method. The cutting orchard was established by the cutting seedlings from 18-year-old elite plants.

### The pre-treatment of explants

In August 2015, after cutting, the shoots were immediately treated as follow: cleaning with a soft brush, soaking in washing powder water for 30 min, and rinsing with water for 10 min. Then we cut the shoots into 2–3 cm sections, and washed with water for 1 h, and finally explants were ready to use.

### The types and concentrations of antimicrobial agents

Carbendazim and Chlorothalonil were selected as bacteriostatic agents. Four concentrations (0.2 g L^−1^, 0.4 g L^−1^, 0.6 g L^−1^, 0.8 g L^−1^) were set for the two antibacterial agents. After pre-treatment, the explants were inoculated in the mediums with different concentrations of bacteriostatic agents and agar (7 g L^−1^). 24 explants were used for each treatment and repeated three times.

### The types and disinfection of explants

According to the previous studies [15, 16, 20, 25], following three types of explants were used: the upper part (the shoot section with upper first and second axillary bud, and having leaves) and middle part (the shoot section with third to fifth axillary bud) for two types of explants, including with and without leaves. The explants were disinfected as the protocol listed in Table 5 after pre-treatment, and then 21 explants of each type were inoculated in the medium (containing Chlorothalonil 0.2 g L^−1^ + agar 7 g L^−1^), and repeated three times.

**Table 5:** Explant type and disinfection strategy for bud induction of *Acacia auriculiformis*

Explant type	Disinfection strategy
Upper shoot section with leaves	0.8 g L^−1^ Carbendazim for 1 min
	75% Alcohol for 2 s
	75% Alcohol for 5 s
Middle shoot section with leaves	0.8 g L^−1^ Carbendazim 3 min
	75% Alcohol for 10 s
	75% Alcohol for 15 s
Middle shoot section without leaves	0.8 g L^−1^ Carbendazim 3 min
	75% Alcohol for 10 s
	75% Alcohol for 15 s

### Medium of bud induction

Modified MS (large elements of MS reduced to half), 1/2 MS (all elements of MS were halved), 1/4 MS (all elements of MS were 1/4), 1/8 MS (all elements of MS were 1/8), and control (no medium) mediums were used. Three levels of 6-BA, including 0 mg L^−1^, 0.5 mg L^−1^, and 1.0 mg L^−1^, were used. After pre-treatment, 24 best explants were inoculated with the different types of mediums that contained various concentrations of 6-BA (containing Chlorothalonil 0.2 g L^−1^ + agar 7 g L^−1^), and each treatment was repeated three times.

Further, different concentrations of 6-BA (0.5 mg L^−1^, 1.0 mg L^−1^, 1.5 mg L^−1^ and 2.0 mg L^−1^) were added in 1/8 MS (containing Chlorothalonil 0.2 g L^−1^ + agar 7 g L^−1^) to screen out the suitable concentration of 6-BA for bud induction (Fig. 1). In total, 24 explants were used for each treatment and repeated three times.

### Explants collection season

The explants were collected in January, April, July and October by using the best induction medium and screened out the best season for explants collection to induce the bud under open conditions. 72 explants were collected for each season and each experiment was repeated three times.

### Culture condition

All of the above experiments were carried out under open condition (non-axenic) i.e. without using ultra-clean bench. All culture mediums were sugar-free, and did not need high temperature or high pressure sterilization. The growth status of explants for each trial was recorded after culturing for 15 day, 30 day and 45 day. The pH value of medium was adjusted to 6.0 before the antibacterial agent was added. The light duration was 12 h day^−1^ and the light intensity was 2500 Lx.

### Data analysis

Differences among treatments were determined by variance analysis and the least significant difference method (LSD) with SPSS19.0. The parameters were recorded as follow:

- **Pollution rate** (%) = Number of polluted explants/Number of inoculated explants × 100
- **Browning rate** (%) = Number of browning explants/Number of inoculated explants × 100
- **Survival rate** (%) = Number of survived explants/Number of inoculated explants × 100
- **Bud induction rate** (%) = Number of explants with bud induction/Number of survived explants × 100.
